# Rapid multi-task diagnosis of oral cancer leveraging fiber-optic Raman spectroscopy and deep learning algorithms

**DOI:** 10.3389/fonc.2023.1272305

**Published:** 2023-10-10

**Authors:** Xing Li, Lianyu Li, Qing Sun, Bo Chen, Chenjie Zhao, Yuting Dong, Zhihui Zhu, Ruiqi Zhao, Xinsong Ma, Mingxin Yu, Tao Zhang

**Affiliations:** ^1^ Department of Stomatology, Peking Union Medical College Hospital, Chinese Academy of Medical Sciences and Peking Union Medical College, Beijing, China; ^2^ Key Laboratory of the Ministry of Education for Optoelectronic Measurement Technology and Instrument, Beijing Information Science and Technology University, Beijing, China; ^3^ Department of Plastic Surgery, Peking Union Medical College Hospital, Chinese Academy of Medical Sciences and Peking Union Medical College, Beijing, China; ^4^ Department of Pathology, Peking Union Medical College Hospital, Chinese Academy of Medical Sciences and Peking Union Medical College, Beijing, China; ^5^ Plastic Surgery Hospital, Chinese Academy of Medical Sciences and Peking Union Medical College, Beijing, China

**Keywords:** Raman spectroscopy, oral cancer, TNM classification, histological diagnosis, machine learning algorithm

## Abstract

**Introduction:**

Oral cancer, a predominant malignancy in developing nations, represents a global health challenge with a five-year survival rate below 50%. Nonetheless, substantial reductions in both its incidence and mortality rates can be achieved through early detection and appropriate treatment. Crucial to these treatment plans and prognosis predictions is the identification of the pathological type of oral cancer.

**Methods:**

Toward this end, fiber-optic Raman spectroscopy emerges as an effective tool. This study combines Raman spectroscopy technology with deep learning algorithms to develop a portable intelligent prototype for oral case analysis. We propose, for the first time, a multi-task network (MTN) Raman spectroscopy classification model that utilizes a shared backbone network to simultaneously achieve different clinical staging and histological grading diagnoses.

**Results:**

The developed model demonstrated accuracy rates of 94.88%, 94.57%, and 94.34% for tumor staging, lymph node staging, and histological grading, respectively. Its sensitivity, specificity, and accuracy compare closely with the gold standard: routine histopathological examination.

**Discussion:**

Thus, this prototype proposed in this study has great potential for rapid, non-invasive, and label-free pathological diagnosis of oral cancer.

## Introduction

According to the Global Cancer Statistics 2020 (GLOBOCAN 2020), both the incidence and mortality of cancer worldwide has been steadily increasing. This increase is indicated by the 377,713 new cases and 177,757 deaths attributed to oral and lip tumors in 2020 (1). The global incidence of oropharyngeal cancer is on the rise (2), and oral tumor prevalence is remarkable escalating in certain developing nations (3). Notably, oral squamous cell carcinoma (OSCC) constitutes over 90% of all oral tumor cases (4). The primary risk factors for oral cancer include smoking, alcohol consumption, betel nut consumption, sun exposure, and HPV infection (5–7). Despite advancements in treatment methods for oral tumors—ranging from surgery and radiation to chemotherapy, immunotherapy, and targeted therapy (8)—the five-year survival rate for oral squamous cell carcinoma has remained below 50% for the past three decades (9). However, early-stage tumor patients experience a significant improvement in the five-year survival rate post-effective treatment (10). Thus, early diagnosis and treatment are pivotal in enhancing the survival rate of oral tumor patients and minimizing mortality (11). Regrettably, most early-stage oral tumor patients often delay treatment due to misdiagnoses, often mistaken for oral ulcers or chronic inflammatory changes, as early oral tumors closely resemble benign lesions (12, 13). Consequently, physicians struggle to accurately distinguish them via visual inspection and palpation (14). Biopsy, though considered the diagnostic gold standard for oral tumors, presents challenges—it is time-consuming, invasive, costly, and demands significant diagnostic skill from pathologists (15). This results in delayed diagnosis and referrals, leading to treatment postponement and reducing survival time for many oral tumor patients (16). Additionally, patients with differing levels of oral cancer pathological differentiation display marked variance in prognosis (17). Research reveals that the five-year survival rate is 89% for patients with well-differentiated oral cancer, compared to 68% and 45% for those with moderate-differentiation or poor-differentiation respectively (18). Hence, the development of a new technology for quick, real-time, portable, and non-invasive diagnosis of oral tumors—capable of providing personalized optimal treatment plans and prognostic information—would enhance diagnostic efficiency and increase patient survival rates.

As illustrated in **Table 1**, Raman spectroscopy technology has become a prevalent tool in the pathological diagnosis research of oral tumors. Micro-Raman spectroscopy, a prominent technique within the realm of Raman spectroscopy, has proven instrumental in distinguishing between benign and malignant formations in oral tumor tissue analyses. This is predominantly achieved through the analysis of hematoxylin and eosin (H&E) tissue sections (19), frozen sections (20), and ex-vivo tissues (21), among other samples. Furthermore, surface-enhanced Raman spectroscopy (SERS) facilitates the diagnosis of oral tumors by analyzing biological specimens such as saliva (22) and serum (23, 24) from patients afflicted with the condition. Up until now, there has been a limited number of studies utilizing Raman spectroscopy for the precise diagnosis of tumor-node-metastasis (TNM) staging and identifying pathological grades such as well differentiated (Grade I), moderately differentiated (Grade II), and poorly differentiated (Grade III). Numerous research groups have successfully employed Raman spectroscopy in analyzing tissue samples or cell lines from patients suffering from diseases like breast cancer (29), brain cancer (30), esophageal squamous cell carcinoma (31), and bladder cancer (32), yielding significant findings. These groups have devised various models leveraging specific algorithms to accurately discern tumor stages or pathological classifications. Sharma et al. (21) have analyzed the intrinsic molecular changes in tissues at different T-stages of oral cancer patients using microscopic Raman spectroscopy technology and have established a diagnostic model for healthy tissues and malignant tumor tissues. However, this study has not yet developed a multi-task diagnostic model that can simultaneously predict the T-stage, N-stage, and pathological grades of oral tumor patients. Xue et al. (25) utilized Surface Enhanced Raman Spectroscopy (SERS) to analyze the serum samples of patients with oral squamous cell carcinoma, thereby predicting their tumor stage and pathological status, with an accuracy of only 85%. However, this method also faces many challenges, such as invasiveness, dependence on external reagents, time-consuming, and complex procedures. Fiber-optic Raman spectroscopy technology circumvents existing hurdles, promising a notable enhancement in diagnostic precision. Singh et al. (26) previously applied this technique to *in-vivo* assessments of individuals with oral tumors, successfully facilitating the pathological grading of normal, precancerous, and tumor tissues. Concurrently, Aaboubout et al. (27) analyzed freshly removed tissues, distinguishing between benign and malignant samples with a sensitivity of 85% and a 92% accuracy rate. Our prior research underscored the potential of portable fiber-optic Raman spectroscopy (PFORS), in conjunction with various machine learning algorithms, to differentiate between cancerous and adjacent tissues in patients afflicted with gum and cheek cancers (28). Nevertheless, the integration of this research into clinical applications has been hampered primarily due to the limited scale of existing research datasets, the challenging nature of ensuring model generalizability, and the protracted duration required for data compilation. Paramountly, before being sanctioned for clinical utilization, these devices necessitate meticulous clinical trials and adherence to medical device safety standards.

**Table 1:** Raman Spectroscopy studies on oral cancers.

Author	Sample type	Raman type	Research results
Meksiarun (19)	H&E tissue sections	Micro-raman	Tumor tissue analysis
Froukje (20)	Frozen sections	Micro-raman	Benign and malignant identification
Sharma (21)	Ex-vivo samples	Micro-raman	Benign and malignant identification
Borsa (22)	Saliva samples	SERS	Benign and malignant identification
Amber (23)	Blood serum samples	SERS	Benign and malignant identification
Moisoiu (24)	Blood serum samples	SERS	Benign and malignant identification
Xue (25)	Blood serum samples	SERS	Tumor stages and pathological status identification
Singh (26)	*In-vivo* detection	Fiber-optic Raman	Benign, pre-malignant and malignant identification
Aaboubout (27)	Fresh resection samples	Fiber-optic Raman	Benign and malignant identification
Chang (28)	Fresh resection samples	Fiber-optic Raman	Benign and malignant identification

This research utilizes a portable fiber-optic Raman spectrometer to investigate ex vivo tissues from oral cancer patients, aiming to determine TNM staging and assess the histologic status. Given the high variability in tumor type, location, and histological grading among oral cancer patients, the tumor specimens exhibit marked heterogeneity, leading to relatively unstable Raman spectroscopic data (33, 34). Consequently, our objective is to provide a comprehensive representation of the spectral characteristics of oral tumors. We accomplish this by expanding the patient sample size, procuring spectral data from diverse anatomical sites, and gathering a substantial dataset of Raman spectroscopic data. In parallel, to unveil hidden features of the Raman spectra and subsequently ascertain the TNM staging and pathologic grading of patients, we have engineered a multi-output deep learning model for spectral data analysis and multi-task network (MTN) classification. Thus, this paper’s primary contributions are outlined below (1): the development of a portable prototype for Raman spectroscopy (2); the creation of a MTN Raman spectroscopy classification model, capable of concurrently diagnosing tumor stages and pathologic grades upon the extraction of shared backbone network features (3); the application of the model to perform a comprehensive visualization analysis and molecular feature interpretation of Raman spectroscopy data.

## Materials and methods

### The portable fiber optic Raman spectrometer prototype

**Figure 1:**
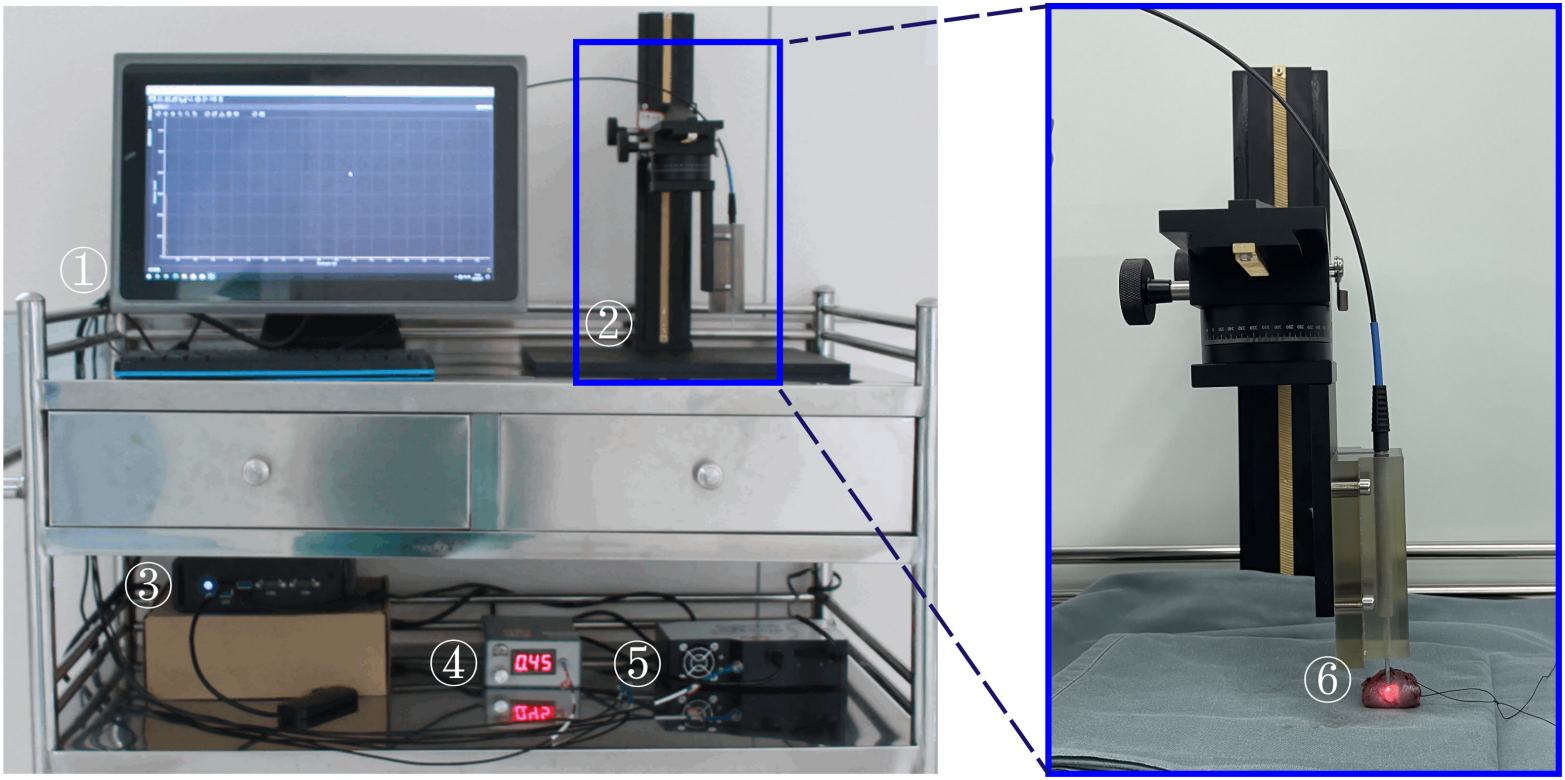
Portable Fiber-optic Raman Spectrometer (PFORS) Prototype. This system includes: ① display; ② manual displacement platform; ③ Edge computing device; ④ diode laser; ⑤ spectrometer; ⑥ fiber-optic Raman spectroscopy probe.

The research employs a portable fiber-optic Raman spectrometer (PFORS) prototype developed by our team for the collection of spectral data from oral cancer tissues, as illustrated in **Figure 1**. A diode laser, employing a fiber coupling of 785 nm, serves as the excitation source. This is introduced through the handheld fiber Raman probe (HT-PROB-MULTI-785, Emvision, LLC), which is linked to fiber-optic cables to facilitate laser excitation, and standard fiber for signal acquisition (NA = 0.22), providing a resolution of 6 cm^−1^. The Raman signal is gathered using a fiber spectrometer (QE65 Pro, Ocean Optics, USA), relying on a charge-coupled device (CCD). Throughout the detection process, the CCD operates at a temperature of -20°C. The spectrometer communicates with the PC through the OceanView interface.

### Patient enrollment and sample preparation

A total of 36 patients with oral cancer who met the inclusion criteria received surgical treatment at Peking Union Medical College Hospital between 2022 and 2023. Comprehensive data including age, gender, and diagnosis were meticulously documented prior to surgery. We acquired the test samples from 48 surgically excised tissues of the participating patients. To prevent contamination that could disrupt spectral signals, any blood stains on the surface of the test samples were meticulously rinsed with running water before conducting fiber Raman spectroscopy. Fresh samples were examined through fiber Raman spectroscopy within 30 minutes post-surgery. After completing the detection process, the localized diseased tissues, and adjacent healthy tissues, for which spectra had been detected, were collected. These samples were then fixed in formalin, embedded in paraffin, and stained with hematoxylin and eosin. An experienced clinical pathologist then performed diagnoses on the pathological sections, assessing disease type, stage, and degree of differentiation. In this study, those examining the Raman spectra of the test tissues, as well as the pathologists conducting pathological testing of the tissues, were blinded to the Raman spectra.

### Data acquisition

In Raman spectroscopy detection, the fiber-optic Raman spectroscopy probe maintains a distance of 0.5 mm from the surface of the tissue under test to ensure stable spectral signals. The measurement of distance is facilitated through visual estimation aided by a flat ruler with a thickness of 0.5mm produced via 3D printing. Prior to every measurement, the background spectrum undergoes an automatic subtraction with a one-second integration time while the laser is deactivated. Subsequently, with the laser reactivated, 90 measurements are conducted on both the tumor and normal tissue surfaces, each with a one-second integration time. The laser power at the probe tip is meticulously calibrated to 100 mW cm^−2^. The entire detection procedure is assuredly concluded within 30 minutes post tissue excision.

### Multi-task network model

#### Data pre-processing

Initially, we categorized all the Raman spectroscopy data by type and selected the spectroscopy data within the range of 400 - 1400 cm^-1^ for further analysis. At the same time, in order to solve the problem of unbalanced sample classes, we average the spectra of samples with a relatively large number of categories. Specifically, for healthy spectra, we average five spectra into one spectrum; For high-T1-N0 tissue spectra, we averaged the three spectra into one spectrum; For the tissue spectra of high-T2-N0 and high-T2-N1, we averaged the five spectra into one spectrum. For the tissue spectra of high-T4-N0 and high-T4-N1, we averaged the six spectra into one spectrum. At the same time, we also removed part of the Raman spectral data that were obviously abnormal. To mitigate the impact of robust fluorescence signals and noise originating from disparate background sources, it was essential to preprocess the spectroscopy data prior to its analysis. This preprocessing phase comprised of three steps:

(i) The Savitzky-Golay filter is utilized during signal denoising to smoothen the spectral data, hence reducing the effect of noise on the spectrum.
(ii) Following signal denoising, the least squares method is employed for baseline correction to fit the polynomial baseline and eliminate the fluorescent background from the initial spectrum.
(iii) Lastly, data normalization is executed using minimum-maximum intensity normalization to standardize the intensity of all spectra within the [0,1] range, facilitating comparison among various samples.

#### Model architecture

**Figure 2:**
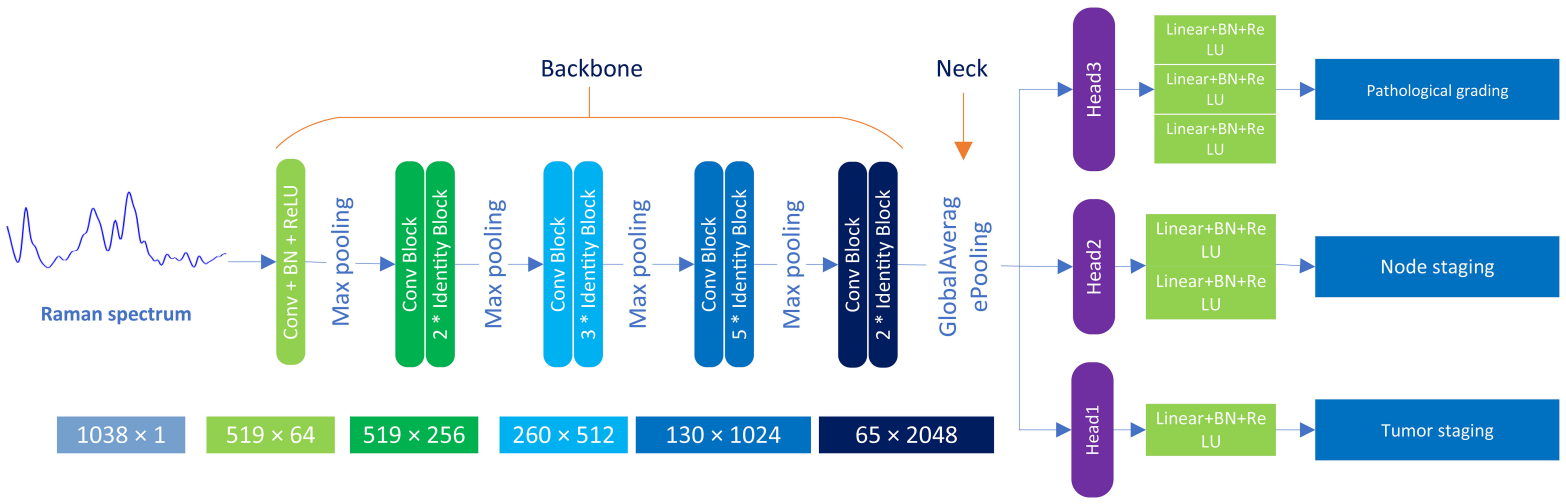
Architecture of MTN-ResNet50 model. The model employs Raman data, comprised of 3534 individual spectra, which are separately introduced to the Backbone, Neck, and Head modules of the system. The Backbone module is primarily responsible for feature extraction, employing 5 distinct convolution layer modules and 12 Identity Blocks. The Neck module undertakes a global average pooling process, whereas the Head module handles the classification task, with the inclusion of 6 fully connected layers.

This paper presents the MTN-ResNet50 model, designed to concurrently perform tumor staging, lymph node staging, and histological grading of oral cancer tissues. The architecture of this network, as illustrated in **Figure 2**, consists of three distinct components: the Backbone, the Neck, and the Head. The Backbone component employs the ResNet50 network structure (35) to extract the feature information from the Raman spectroscopy of oral cancer tissues. This backbone comprises 5 convolutional modules, 12 identity blocks, and 4 pooling layers, collectively forming a five-layer network structure. In particular, the initial layer of the network employs 64 convolutional kernels with dimensions of 7×1 and incorporates a 3×1 Max pooling operation. This operation results in an output image size of 519 x 64. Subsequently, the second through fifth layers are composed of residual modules, each generating feature maps with dimensions of 519×256, 260×512, 130×1024, and 65×2048, respectively. The Neck component is responsible for processing the extracted spectroscopic data and employs adaptive global average pooling to pool the input data, transforming it into a suitable format for subsequent processing. Lastly, the Head component handles the classification tasks for tumor staging, lymph node staging, and histological grading. It utilizes three classification head, contains a total of six full connection layer to generate classification results for the three tasks at the same time. Specifically, we have designed three separate classifiers within the head component to cater to different tasks, including tumor staging, lymph node staging, and histological grading classification. For the tumor staging task, we used three fully connected layers for building a seven-class model. Similarly, a five-class model was constructed using two fully connected layers for lymph node staging, and a single fully connected layer was utilized for histological grading, leading to a five-class model.

#### Model training

To achieve the final classification, we employed the Softmax for calculating the probability of the outputs. The learning rate is set to 0.0001, a batch size is set to 256, and training epochs are set to 1000. The stochastic gradient descent algorithm was used for optimization, with a momentum of 0.9 and a weight decay of 0.00002.

To test the model’s generalizability, we randomly partitioned the dataset into ten subsets. During each training session, seven subsets were used for training, two for validation, and the remaining one was reserved for testing. This process was repeated ten times, each time using a different subset for testing. Finally, the mean of the ten models’ evaluation results served as the model’s performance metric.

To provide an easily interpretable explanation of the MTN-ResNet50 model’s performance in Raman spectroscopy data analysis, we used the Gradient-weighted Class Activation Mapping (Grad-CAM) approach (36). The CAM analysis enabled us to visualize the Raman spectral regions that the MTN-ResNet50 model was focusing on, thereby facilitating a better understanding of the classification process. Initially, the spectral data was fed into the MTN-ResNet50 model, and the Grad-CAM method was used to calculate and plot the gradients of the last convolutional layer’s feature map. Subsequently, these gradients were weighted and summed with the feature map from the last convolutional layer, followed by global average pooling to obtain a heatmap corresponding to the target class.

Lastly, the heatmap and the original spectral image were superimposed on a single graph. This approach provided an intuitive visualization of spectral differences across bands and aided in our comprehension of spectral data characteristics and variations.

## Result

### Flowchart

**Figure 3:**
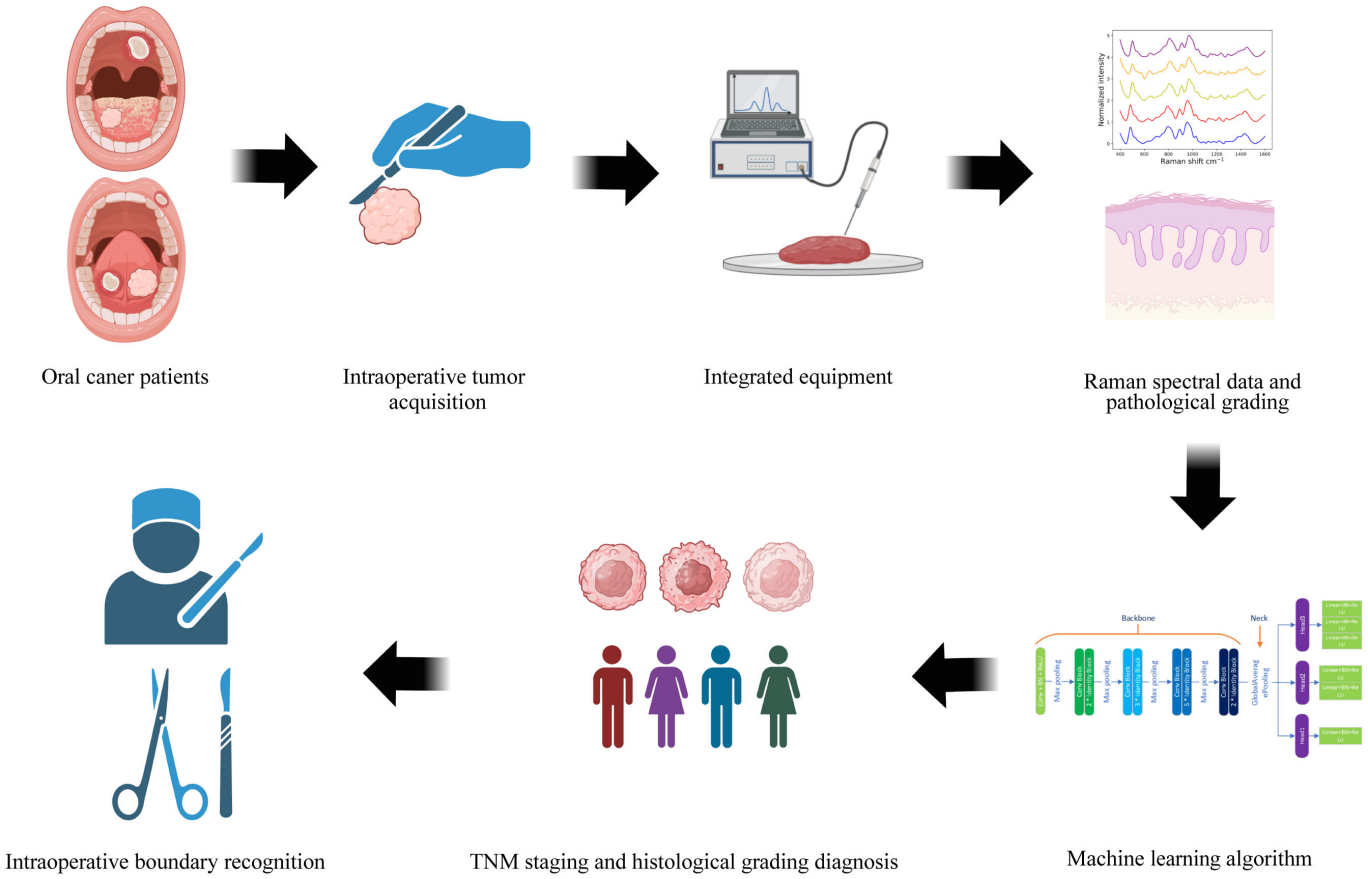
The flowchart depicts the pathological staging and histological grading diagnosis process for oral cancer tissue, leveraging fiber-optic Raman spectroscopy technology and deep learning algorithms.

**Figure 3** provides a schematic diagram depicting the workflow for tumor staging and histological grading of oral cancer patients, utilizing Raman spectroscopy techniques and deep learning algorithms. This figure was generated using BioRender.com. Initially, oral tumor patients are recruited, and the relevant tissues for examination are harvested during surgery. We then acquire Raman spectroscopy data from both the oral tumor and the tissue adjacent to it. Concurrently, we collect essential demographic information about the patients enrolled and the pathological diagnosis corresponding to the tissues under examination. Subsequently, this spectral input is integrated into a MTN model designed for diagnosing oral cancer pathologically, thereby facilitating real-time diagnostic assessment of the cancer’s stage and its pathological progression using this device. Ultimately, the potential application of this portable fiber-optic Raman spectrometer lies in its ability to identify intraoperative tumor boundaries, thereby providing surgical guidance.

### Patient information

**Table 2** outlines the patient characteristics for this study. Patients with oral cancer are categorized into five groups (Tis, T1, T2, T3, T4) based on tumor size and extent of tumor involvement. The study also groups patients into N0, N1, and N2 based on lymph node metastasis and its features. Patients who fall under N3 classification or exhibit distant metastasis, for whom further surgical intervention is not recommended, are excluded from this study. Patients’ histological diagnosis is categorized as health, benign tumor or dysplasia (BOD), well differentiated (WD), moderately differentiated (MD) and poorly differentiated (PD) tissue, according to the World Health Organization’s histological classification (37).

**Table 2:** Summaries of the fundamental information for the enrolled oral tumor patients and the corresponding number of collected Raman spectra.

Patient information		Number of patients or samples	Number of spectra
Age	<60year	8	—
	>60year	28	—
Gender	male	23	—
	female	13	—
Test site	tongue	20	—
	Cheek	12	—
	Gingiva	9	—
	Mouth floor	5	—
	Lip	2	—
T-staging	Tis	3	270
	T1	8	720
	T2	15	1350
	T3	2	180
	T4	11	990
N-staging	N0	28	2520
	N1	7	630
	N2	4	360
Histological grading	health	39	3510
	BOD	9	810
	WD	31	2790
	MD	5	540
	PD	3	300

### Raman spectroscopy analysis

#### Results of Raman spectroscopy analysis across various T-staging

**Figure 4:**
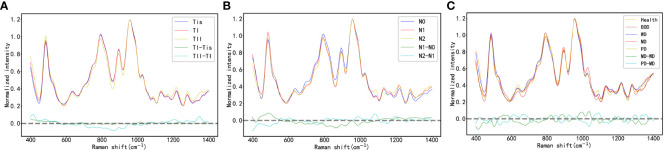
**(A)** the Raman Spectra of Oral Cancer Tissues Across Varying T-Stages; **(B)** the Raman Spectra of Oral Cancer Tissues Across Varying N-Stages; **(C)** the Raman Spectra of Oral Cancer Tissues Across Varying Histological Grades.

The study classifies the 2127 Raman spectroscopy data points from all oral cancer patients into five groups: Tis, T1, T2, T3, and T4, comprising 270 Tis, 300 T1, 432 T2, 180 T3, and 945 T4, respectively. Given the close correlation between T1 and T2, and T3 and T4 in clinical practice and disease management, these categories will be merged for analysis, resulting in two groups: TI and TII. The comparison of Raman spectra between TI and Tis, as well as between TII and TI, reveals distinct differences, detailed in **Table 3**. In **Figure 4A**, an integrated analysis of these differential Raman spectra indicates increased peak values at 484 (Glycogen), 525 (proteins), 1220 (Amide III) cm^-1^, and decreased spectral peaks at 585 (OH out of plane bending), 858 – 863 (Tyrosine, collagen type I) cm^-1^. These peak shifts primarily involve components such as sugars, Amide III, and collagen type I (38, 39).

**Table 3:** Analysis of peak positions and assignment in raman spectra during TI-Nis and TII-TI progressions.

Progression type	Change direction	Raman shift (cm^−1^)	Band assignments
TI-Nis	Increase	685	DNA bases
		1014	Carbohydrates
		1231	Amide III
	Decrease	585	OH out of plane bending and phosphate of HA
		799	Phosphates
		858	Tyrosine, collagen
		983	Lipids
TII-TI	Increase	484	Glycogen
		1206	Hydroxyproline
		1220	Amide III
	Decrease	767	Pyrimidine ring breathing mode
		863	Phosphatidic acid

#### Results of Raman spectroscopy analysis across various N-staging

This study organizes the 2127 Raman spectroscopy data points from all oral cancer patients into three groups: N0, N1, and N2, including 1305 N0, 282 N1, and 540 N2 data points, respectively. By contrasting these groups, we can delineate the differential Raman spectra between N1 and N0, and between N2 and N1 in **Table 4**. As shown in **Figure 4B**, a comprehensive analysis of these differential Raman spectra reveals an increase in peak values at 1174 (phenylalanine), 1195 (Nucleic acids), 1198 (tryptophan) cm^-1^, and a decrease at 728 (collagen), 717-719 (lipids), 719 (phospholipids) cm^-1^. These variations in peak Raman shifts primarily correspond to lipids, tryptophan, phenylalanine, and collagen (38, 39).

**Table 4:** Analysis of peak positions and assignment in raman spectra during N1-N0 and N2-N1 progressions.

Progression type	Change direction	Raman shift (cm^−1^)	Band assignments
N1-N0	Increase	489	Glycogen
		1210	Phenylalanine
		1304	Adenine, cytosine
		1366	Tryptophan
	Decrease	815	Tyrosine, proline, hydroxyproline,
		862	Phosphate group
		983	Lipids
N2-N1	Increase	607	Glycerol
		988	Proteins
		1080	Phospholipids and phosphate vibrations
		1195	Nucleic acids and phosphates
	Decrease	756	Tryptophan
		1151	Carotenoid
		1260	Lipids

#### Results of Raman spectroscopy analysis across various histological grades

**Figure 5:**
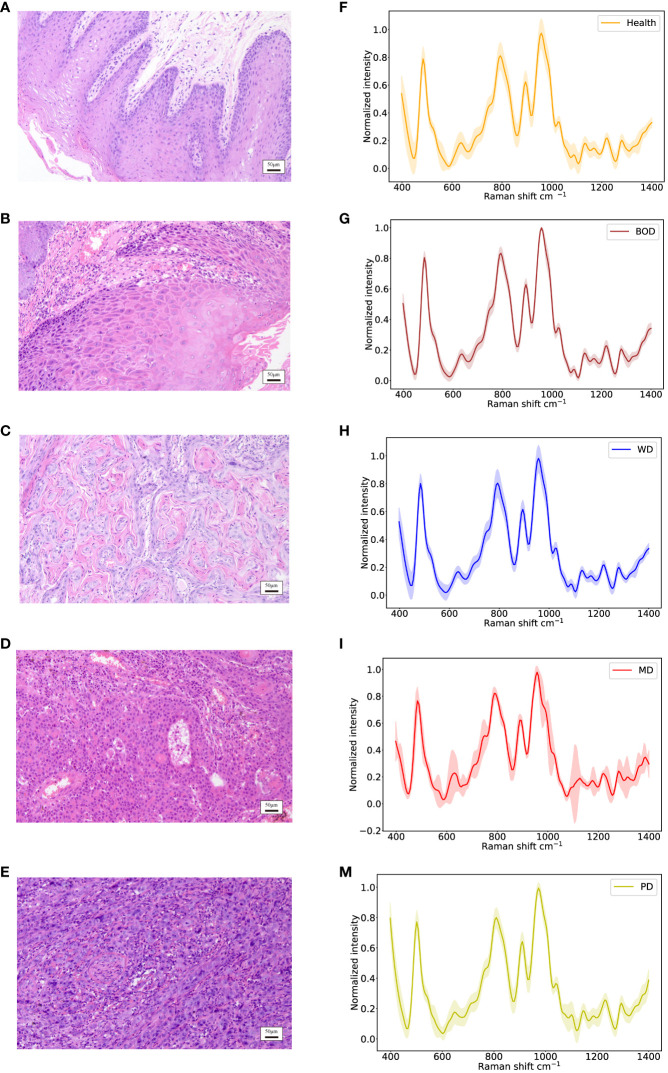
Identification of different histopathologic grades of oral tissues, including Health, BOD, WD, MD, and PD tissues. **(A–E)** Histopathological images; **(F–M)** Raman spectra of different histopathologic grades of oral tissues.

In accordance with pathological classifications, this study divided the 3534 spectral datasets obtained from all the tested tissues into five categories: healthy tissue, benign tumor or dysplasia (BOD), well differentiated (WD), moderately differentiated (MD) and poorly differentiated (PD) tissue, comprising 703, 704, 987, 540, and 600 samples respectively. The pathological diagrams and average Raman spectral datasets for these categories are depicted in **Figures 5A–M**. Spectral charts from patients with the same disease stage and pathologic grade, albeit from different oral cancer patients, showed remarkable similarity, implying significant homogeneity within identical test tissue types. In contrast, spectral data from patients with varying disease stages and pathological grades displayed notable differences. A comparison of Raman spectral data across different pathologic grades revealed significant variation in Raman peaks within specific areas in **Table 5**: during the transition from WD to MD, peak intensities at 820, 889, 998, 1034 cm^-1^ experienced an increase, while those at 501, 1299, 1332 cm^-1^ showed a decrease. Similarly, during the transition from MD to PD, peaks at 613, 1090, 1356 cm^-1^ increased, whereas those at 534, 1034, 1146, 1255 cm^-1^ decreased. Upon conducting a comprehensive analysis of these divergent Raman spectra in **Figure 4C**, both sets showed an increase in Raman spectra at peaks of 815 (nucleic acid), 820 (structural protein modes of tumors), 970 (proteins and nucleic acids), and 1370 cm^-1^ (the most pronounced saccharide band); conversely, peaks at 516 (phosphatidylinositol), 1146 (carbohydrates), 1223 (collagen I), and 1318 cm^-1^ (protein and Amide III) decreased. As indicated by previous studies, these alterations in peak values of the Raman shifts primarily correspond to nucleic acids, structural proteins, and collagen I within tumor cells (38, 39).

**Table 5:** Analysis of peak positions and assignment in raman spectra during MD - WD and PD - MD progressions.

Progression type	Change direction	Raman shift (cm^-1^)	Band assignments
MD - WD	Increase	820	Protein band and structural protein modes of tumors
		889	Methylene rocking
		998	C-O ribose
		1034	Phenylalanine of collagen
	Decrease	501	Methoxy group
		1299	Lipid
		1332	C3-C3 stretch and C5-O5 stretch CHa in-plane bend
PD - MD	Increase	613	Cholesterol ester
		1090	Phosphate
		1356	Guanine
	Decrease	534	Cholesterol ester
		1034	Phenylalanine of collagen
		1146	Carbohydrates
		1255	Lipids

### Result of multi-task network model

**Figure 6:**
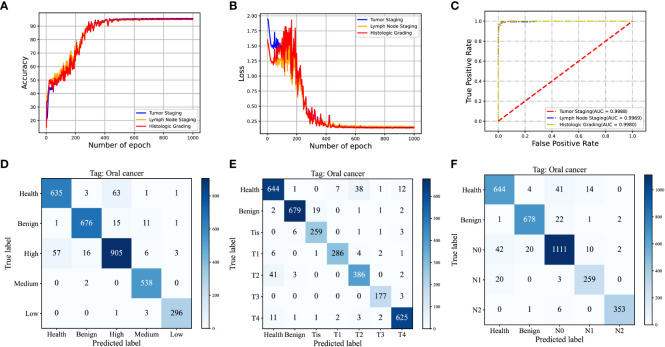
**(A, B)** Validation Accuracy and Cross-Entropy Loss Curves in Iterative Training of Convolutional Neural Networks for T-staging, N-staging, and Histological Grading; **(C)** ROC Curve and Corresponding AUC Values of the Test Set; **(D)** Cumulative Confusion Matrix from Ten-Fold Cross-Validation of the T-Staging Classification Task; **(E)** Cumulative Confusion Matrix from Ten-Fold Cross-Validation of the N-Staging Classification Task; **(F)** Cumulative Confusion Matrix from Ten-Fold Cross-Validation for Histological Grading.

In assessing the performance and reliability of CNN models, accuracy and cross-entropy loss are commonly used metrics. As the learning iterations progress, the accuracy and cross-entropy loss curves of the validation set gradually converge, indicating that the model does not suffer from overfitting. **Figures 6A, B** present the accuracy curves and cross-entropy loss curves for the three subtasks depicted in this study.

For comparison purposes, we opted for VGG16 and Support Vector Machines (SVM) as benchmark models. VGG16 (40), a well-known Convolutional Neural Network (CNN), is recognized for its remarkable feature extraction capability. In order to achieve multi-task learning, we made modifications to the VGG16 network model and created another multi-task network model called MTN-VGG16, aimed at adapting to multiple classification tasks such as tumor staging, lymph node staging, and histological grading. On the other hand, SVM, a conventional machine learning algorithm, is extensively applied in classification tasks. We used a one-versus-all strategy to realize MTN learning with SVM. Specifically, we treated the three classification tasks as independent and processed them by sequentially training and testing three SVM classifiers.

**Table 6** illustrates the performance measures, including accuracy, specificity, and sensitivity, of our MTN-ResNet50 model, the MTN-VGG16 model, and the SVM algorithm on the three classification tasks. A comparative analysis reveals that our MTN-CNN model exhibits superior performance across all tasks. Particularly, for the T-stage classification task, our model yielded an accuracy, specificity, and sensitivity of 94.49%, 99.06%, and 94.83% respectively. For the N-stage classification task, these measures were 94.15%, 98.41%, and 94.31% respectively. For the pathologic grading classification task, the measures were 94.30%, 98.48%, and 95.25% respectively.

**Table 6:** Performance of MTN-ResNet50, MTN-VGG16, and SVM algorithms in T-staging, N-staging, and histological grading identification.

Algorithm types	Classification	Accuracy (%)	Specificity (%)	Sensitivity (%)
ResNet50	T-staging	94.49 ± 1.33	99.06 ± 0.24	94.83 ± 1.46
	N-staging	94.15 ± 1.25	98.41 ± 0.35	94.31 ± 0.97
	Histological Grading	94.30 ± 1.30	98.48 ± 0.34	95.25 ± 1.13
VGG-16	T-staging	92.83 ± 1.44	98.77 ± 0.26	92.77 ± 1.43
	N-staging	92.79 ± 1.55	98.01 ± 0.44	92.78 ± 1.55
	Histological Grading	90.85 ± 1.79	97.58 ± 0.47	92.14 ± 1.76
SVM	T-staging	88.43 ± 1.09	97.97 ± 0.20	89.68 ± 1.49
	N-staging	85.62 ± 1.71	95.97 ± 0.51	87.10 ± 1.41
	Histological Grading	86.15 ± 1.56	96.32 ± 0.41	88.11 ± 1.30

To quantitatively assess the performance of the MTN-ResNet50 model, we generated the Receiver Operating Characteristic (ROC) curves for the tumor staging, lymph node staging, and pathologic grading classification tasks, and calculated the Area Under the Curve (AUC). The corresponding AUC values were 0.9971, 0.9931, and 0.9969 respectively as shown in **Figure 6C**. These results corroborate the superior performance of the MTN-ResNet50 model for these tasks. To visually represent the prediction accuracy and error rates of our classification model across different categories, we present the confusion matrices corresponding to the results of the ten-fold cross-validation for the three classification tasks in **Figures 6D–F**. The visual representation of confusion matrices aids in understanding the prediction behavior of the classification model in each category and provides a clear understanding of the model’s classification performance.

### Visual analytical approach to Grad-CAM

**Figure 7:**
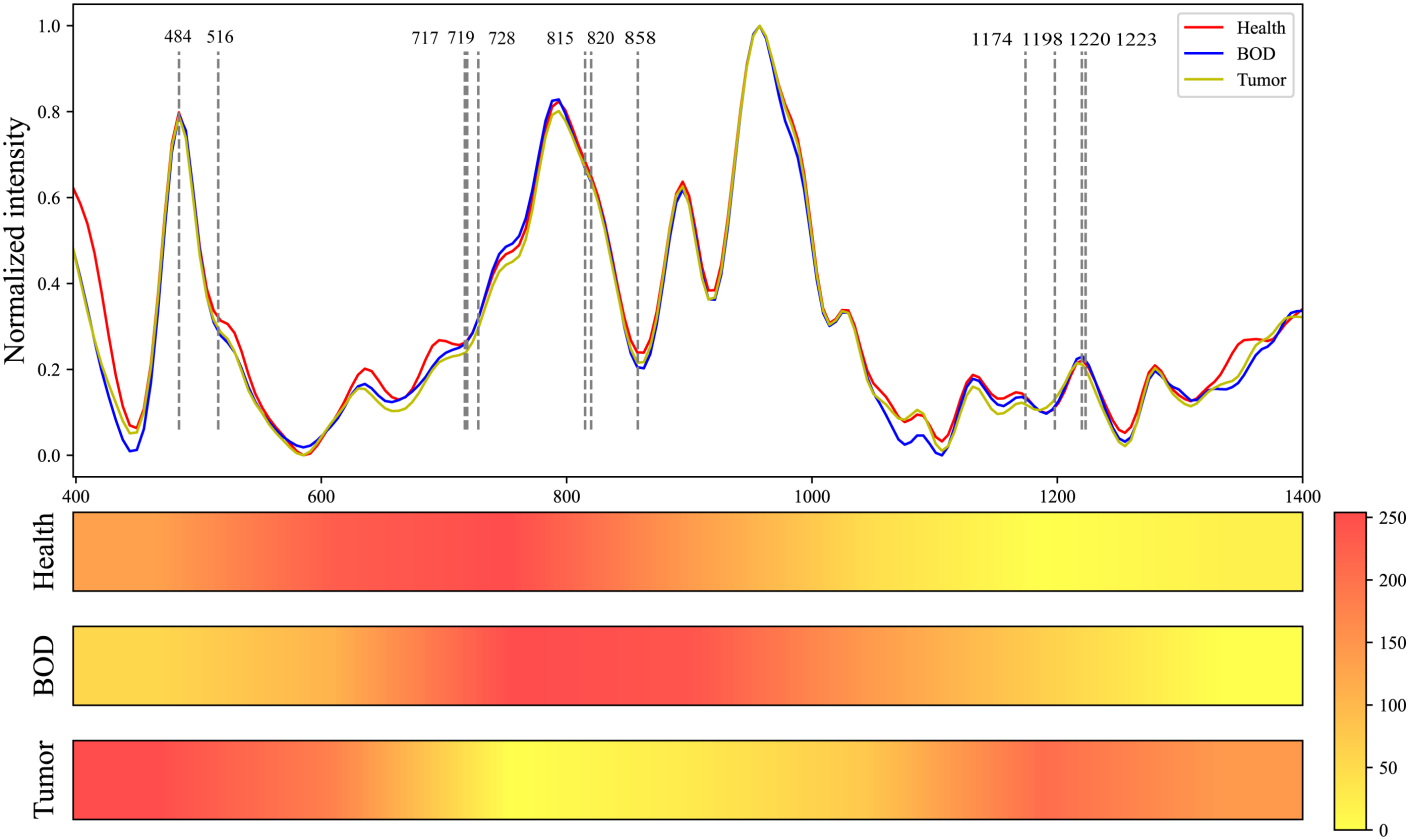
Comparative Raman Spectroscopy Profiles of Healthy, BOD, and Malignant Tissues, Coupled with the Heatmap Visualization of a Grad-CAM Neural Network.

Utilizing the Grad-CAM tool, we generated visualizations of Raman spectra for three categories: healthy tissue, BOD, and malignant tumor tissue using our MTN-ResNet50 model. To facilitate an intuitive comparison between these categories, **Figure 7** presents the average Raman spectra and Grad-CAM neural network heatmap. The color intensity in these visualizations is indicative of the influence that specific area has on the model’s target category prediction, with redder areas demonstrating higher influence and lighter areas suggesting lower influence. By visualizing these Raman spectra, it becomes apparent that the MTN-ResNet50 model places emphasis on different Raman shift areas for different datasets.

More specifically, the model focuses on the spectral range of 542 cm^-1^ to 880 cm^-1^ for healthy tissue, 695 cm^-1^ to 1020 cm^-1^ for BOD, and 400 cm^-1^ to 625 cm^-1^ and 1170 cm^-1^ to 1270 cm^-1^ for malignant tumor tissue. Through these visualizations, we can delineate the variations between different tissue types, thereby enhancing our understanding of Raman spectra classifications. As shown in **Table 7**, several biomolecules have been reported to correlate strongly with our research, and they reside within the Raman shift regions highlighted by Grad-CAM. These biomolecules, which hold the potential to characterize the biochemical features of various biological tissues, enhance the interpretability of our classifications.

**Table 7:** Analysis of peak positions and assignment in raman spectra of healthy, BOD, and malignant tissues .

Raman shift (cm^−1^)	Intensity	Band assignment
484	w, s	Glycogens
516	m, s	Phosphatidylinositol
717-719	s, m	Lipid
719	s, m	Phospholipid
728	s, s	Collagen
815	s, s	Nucleic acid
820	s, s	Structural protein modes of tumors
858–863	m, s	Tyrosine, collagen type I
1174	m	Phenylalanine
1198	s, m	Tryptophan
1223	m	Collagen I
1220	s	Amide III

Intensity: weak (0.4-0.6), mid (0.6-0.8) and strong (0.8-1).

## Discussion

Raman spectroscopy is utilized for the detection and analysis of biochemical components in biological tissues, with primary constituents including proteins, lipids, and nucleic acids (41). Through the measurement of molecular vibrational modes, fiber-optic Raman spectroscopy provides intricate details about the composition and concentrations of these biochemical components (42). The spectral characteristics are principally determined by the biochemical components and histological characteristics of the tested samples (32). This study is the first to extract the biochemical characteristics of oral lesions at different pathological stages and histological grades through fiber-optic Raman spectroscopy combined with deep learning algorithms. Then, a “Spectroscopy-TNM staging-histological grading” model was established using a MTN learning algorithm to predict the pathological diagnosis of oral tumor patients. Previous research teams have distinguished between benign and malignant human tissues by analyzing Raman spectroscopy data and extracting essential spectral characteristics via various machine learning algorithms (43, 44). The multi-output model utilized in this study can concurrently execute multiple tasks, including TNM staging and histological grading. By extracting shared features, the model can glean associative information amongst distinct tasks, thereby enhancing the model’s generalizability.

Raman spectroscopy is instrumental in TNM staging of OSCC patients, as it correlates with the types and concentrations of biochemical constituents within the tissues (45). Studies reveal that shifts in the composition and structure of these biochemical constituents will lead to changes in the signal intensity at different Raman shifts (46). An analysis of Raman spectra from patients at various T-stages, integrated with key areas indicated by Grad-CAM analysis, demonstrates an increase in glycogens and Amide III, as well as a decrease in Tyrosine and Collagen type I between Tis and TI, and between TII and TI. These findings align with prior research asserting that cancer cells require an augmented glucose supply for rapid growth and division (47). Moreover, cancer cells can exhibit alterations in the extracellular glycan structure, utilizing these glycans for immune evasion (48). An observed increase in Amide III may be attributable to the cancer cells’ stress response to inadequate nutrition and oxygen, as exemplified by the elevated expression of heat shock proteins (49). Employing the ResNet50 algorithm, the overall accuracy, specificity, and sensitivity for different T-stages are reported to be 94.88 ± 1.38%, 99.12 ± 0.24%, and 95.23 ± 1.46%, respectively.

In addition, while N-staging greatly impacts the choice of therapeutic approach and patient prognosis, its accurate determination currently necessitates pathological analysis of surgical specimens (50). Prior studies, utilizing Raman spectroscopy, have achieved 100% accuracy in differentiating lymph nodes containing metastatic tumors in breast cancer patients (51, 52). However, no study thus far has predicted lymphatic metastasis via direct tumor examination. Previous *in vitro* experiments indicated Raman spectroscopy’s capability in distinguishing mouse cancer cell lines with varying metastatic potentials and invasiveness (53). In this investigation, Raman spectroscopy revealed a decrease in lipid or fatty acid and phospholipid accumulation, collagen, alongside an increase in Tryptophan and Phenylalanine between stages N1 and N0, as well as between N2 and N1. Literature corroborates that a “low lipid” phenotype in tumor tissues is indicative of enhanced cellular migration *in vitro* and increased metastatic ability *in vivo (* 54). Research has established that MMP-2 and MMP-9 foster tumor invasion and metastasis through collagen degradation, leading to extracellular matrix disruption and consequent cellular dysfunction (54–56). An increase in Tryptophan in cancer tissues, catalyzed by indoleamine 2,3-dioxygenase (IDO) into immunosuppressive guanosine, facilitates immune evasion (57). Nonetheless, a unique observation of sugar reduction at 490 cm^-1^ when transitioning from N2 to N0 remains unexplained within the biological context. However, the correlation between changes in Raman spectroscopy and tumor staging is not constant, primarily due to the fact that tumor staging doesn’t encapsulate the trend of alterations in all biomolecules. Algorithmic analysis has shown that the overall accuracy, specificity, and sensitivity across different N-stages are 94.57 ± 1.32%, 98.54 ± 0.35%, and 94.47 ± 1.47% respectively.

Histopathological identification of OSCC relies on aspects such as cellular morphology and tissue architecture (58). The pathological type of the tumor plays a crucial role in guiding the choice of treatment regimens and in prognostic assessment (59). Our analysis of Raman spectral variations in well, moderately, and poorly differentiated types, augmented with Grad-CAM analysis, revealed a trend consistent with changes in pathological state from WD to PD. Specifically, we observed increased structural protein modes, decreased collagen I, and heightened nucleic acids at spectral positions of 820, 1223, and 815 cm^-1^, respectively. As the malignancy of the tumor escalates, there is an increase in collagen degradation within the tumor, a process known to stimulate angiogenesis, as corroborated by several studies (60). Additionally, as the tumor progresses, tumor-associated fibroblasts primarily responsible for collagen I production undergo phenotypic changes, leading to a decrease in collagen I levels. On another note, to support rapid cellular proliferation and division, cancer cells display amplified nucleic acid metabolism (61). In our study, Raman spectral data analyzed with the ResNet50 algorithm yielded an overall diagnostic accuracy, specificity, and sensitivity for different pathologic grades of 94.34 ± 1.55%, 98.54 ± 0.41%, and 94.96 ± 1.28%, respectively.

This research presents several areas of limitation. Primarily, an imbalance in the sample sizes poses a concern; specifically, some pathological categories lack sufficient sample numbers. Nonetheless, comparable studies have demonstrated effective results through algorithmic processing (62). A secondary limitation lies in the non-application of the model machine *in vivo* due to potential challenges the probe might introduce to surgical sterility requirements (63). Furthermore, the portable fiber-optic Raman spectrometer has yet to be fully integrated, thus impeding true portability. As such, future research by our team will concentrate on component integration to enhance device portability and mobility. Finally, the research is constrained by the recent treatment history of the patients involved, disallowing the collection of prognostic data. As a result, predicting patient prognosis through Raman spectroscopy remains impossible. Nonetheless, it is crucial to acknowledge the potential of this technology in aiding pathologists in faster and more accurate determination of tumor staging and histological grading, thereby reducing diagnostic variability. Once this system accomplishes rapid, label-free, non-invasive, and highly accurate pathological diagnosis, it could facilitate intraoperative tumor boundary diagnosis, and potentially provide significant guidance for preoperative treatment planning and patient prognosis analysis.

## Conclusion

This study demonstrates that fiber-optic Raman spectroscopy can elucidate subtle, real-time changes in the biochemical composition of oral lesion tissues, offering an advantage over traditional histopathological diagnosis. Leveraging this technique in conjunction with machine learning algorithms, we constructed a single pathological diagnosis model that simultaneously achieves MTN diagnosis of oral cancer pathologic staging and histological grading. This is accomplished by extracting shared features across sub-tasks and assimilating related information. Our findings reveal that Raman spectra vary significantly across different pathological stages, reflecting notable changes in the content of glycans, lipids, nucleic acids, and collagen proteins. Raman spectroscopy, as shown in this study, can provide insights into the mechanistic evolution of pathologic grade changes from a biochemical standpoint. Consequently, this technology aids in developing innovative, rapid, non-invasive, and label-free tools for both preoperative and intraoperative pathological diagnosis of oral cancer, which can be applied in outpatient clinics and operating rooms.

## Data availability statement

The original contributions presented in the study are included in the article/supplementary material. Further inquiries can be directed to the corresponding authors.

## Ethics statement

The studies involving humans were approved by Medical Ethics Committee of Peking Union Medical College Hospital. The studies were conducted in accordance with the local legislation and institutional requirements. The participants provided their written informed consent to participate in this study.

## Author contributions

XL: Data curation, Investigation, Methodology, Project administration, Visualization, Writing – original draft. LL: Data curation, Investigation, Methodology, Visualization, Writing – original draft. QS: Investigation, Methodology, Writing – review & editing. BC: Investigation, Methodology, Writing – review & editing. CZ: Investigation, Methodology, Writing – review & editing. YD: Investigation, Methodology, Writing – review & editing. ZZ: Investigation, Methodology, Writing – review & editing. RZ: Investigation, Methodology, Writing – review & editing. XM: Investigation, Methodology, Writing – review & editing. MY: Conceptualization, Funding acquisition, Resources, Software, Supervision, Validation, Writing – review & editing. TZ: Conceptualization, Funding acquisition, Resources, Supervision, Validation, Writing – review & editing.
